# Automatic Transcranial Sonography-Based Classification of Parkinson’s Disease Using a Novel Dual-Channel CNXV2-DANet

**DOI:** 10.3390/bioengineering11090889

**Published:** 2024-08-31

**Authors:** Hongyu Kang, Xinyi Wang, Yu Sun, Shuai Li, Xin Sun, Fangxian Li, Chao Hou, Sai-kit Lam, Wei Zhang, Yong-ping Zheng

**Affiliations:** 1 Department of Biomedical Engineering, The Hong Kong Polytechnic University, Hong Kong SAR, China; hong-yu.kang@connect.polyu.hk (H.K.); haylee-xinyi.wang@connect.polyu.hk (X.W.); sshuai.li@connect.polyu.hk (S.L.); saikit.lam@polyu.edu.hk (S.-k.L.); 2 Research Institute of Smart Ageing, The Hong Kong Polytechnic University, Hong Kong SAR, China; stefanie.sun@polyu.edu.hk; 3 Department of Ultrasound, Beijing Tiantan Hospital, Capital Medical University, Beijing 100070, China; sunxinbrier@foxmail.com (X.S.); Liergou099@163.com (F.L.); houcdoctor@163.com (C.H.)

**Keywords:** Parkinson’s disease, transcranial sonography, auto-classification, deep learning

## Abstract

Transcranial sonography (TCS) has been introduced to assess hyper-echogenicity in the substantia nigra of the midbrain for Parkinson’s disease (PD); however, its subjective and resource-demanding nature has impeded its widespread application. An AI-empowered TCS-based PD classification tool is greatly demanding, yet relevant research is severely scarce. Therefore, we proposed a novel dual-channel CNXV2-DANet for TCS-based PD classification using a large cohort. A total of 1176 TCS images from 588 subjects were retrospectively enrolled from Beijing Tiantan Hospital, encompassing both the left and right side of the midbrain for each subject. The entire dataset was divided into a training/validation/testing set at a ratio of 70%/15%/15%. Development of the proposed CNXV2-DANet was performed on the training set with comparisons between the single-channel and dual-channel input settings; model evaluation was conducted on the independent testing set. The proposed dual-channel CNXV2-DANet was compared against three state-of-the-art networks (ConvNeXtV2, ConvNeXt, Swin Transformer). The results demonstrated that both CNXV2-DANet and ConvNeXt V2 performed more superiorly under dual-channel inputs than the single-channel input. The dual-channel CNXV2-DANet outperformed the single-channel, achieving superior average metrics for accuracy (0.839 ± 0.028), precision (0.849 ± 0.014), recall (0.845 ± 0.043), *F*1-score (0.820 ± 0.038), and AUC (0.906 ± 0.013) compared with the single channel metrics for accuracy (0.784 ± 0.037), precision (0.817 ± 0.090), recall (0.748 ± 0.093), *F*1-score (0.773 ± 0.037), and AUC (0.861 ± 0.047). Furthermore, the dual-channel CNXV2-DANet outperformed all other networks (all *p*-values < 0.001). These findings suggest that the proposed dual-channel CNXV2-DANet may provide the community with an AI-empowered TCS-based tool for PD assessment.

## 1. Introduction

Parkinson’s disease (PD) is a rapidly growing, chronic, progressive, and devastating neurodegenerative disorder that has affected over 9.4 million individuals worldwide as of 2020 [1], and it has been recognized as one of the rapidly advancing neurological disorders [2,3]. Age is the primary risk factor for developing PD, and it is most commonly seen in individuals over the age of 60, with men being more susceptible than women at a prevalence ratio of approximately 3:2 [2,4]. Moreover, various modifiable environmental factors—such as pesticides and water pollutants—as well as lifestyle choices like smoking, coffee consumption, exercise, and head trauma, have all been implicated in the pathogenesis of PD across different populations [5,6]. Identifying the early stages of PD can be challenging, often leading to a significant delay between the emergence of noticeable symptoms and an official diagnosis (averaging around 10 years). Early indicators encompass a spectrum ranging from constipation to a diminished sense of smell, asymmetric shoulder pain, and even symptoms of depression [3]. PD can incur a range of debilitating motor symptoms including tremors at rest, rigidity, akinesia (or bradykinesia), and postural instability [7], as well as non-motor symptoms including autonomic dysfunction, neuropsychiatric symptoms, sensory symptoms, sleep disturbance, etc. [8]. These symptoms have posed grievous consequences to patients, their families and caregivers, and societies. Currently, studies are exploring the diagnosis of PD through various methods including voice, language, movement, and empirical mode decomposition [9,10,11]. However, PD is still considered an incurable condition [12,13]. Treatment aims primarily at slowing or stopping the progression of the disease. Conventional management strategies often initiate with dopamine replacement therapies, such as daily oral administration of carbidopa/levodopa or dopamine agonists [14,15]. Furthermore, regular exercise can enhance neuroprotection, thereby mitigating the advancement of the disease [16,17,18].

A growing body of literature has proven that the release of dendritic dopamine in the substantia nigra (SN) within the midbrain structure is crucial for controlling neuronal activity and behavior [19], and that iron ion accumulation in the SN is closely related to the development of PD and Parkinsonism [20]. With regard to this, the detection of iron ion accumulation in the SN of the midbrain plays a pivotal role in enabling early PD diagnosis [21].

The array of imaging modalities that have been deployed to detect PD [22] includes magnetic resonance imaging (MRI), positron emission tomography (PET), single-photon emission computerized tomography (SPECT), and transcranial sonography (TCS). MRI is radiation-free, has high image resolution, and is capable of quantifying iron ion levels by using specific image acquisition sequences, but it is of low accessibility and affordability to the general public, particularly in developing or under-developed countries, rendering it unfit for the population-wide screening of PD [23]. PET and SPECT imaging are capable of assessing striatal dopamine terminal dysfunction that supports the diagnosis of PD [24]. But they are costly, have low accessibility, and induce radiation hazards in patients. In contrast, TCS presents several distinct advantages over MRI, PET, and SPECT. This modality delivers a non-invasive, radiation-free, real-time, and rapid imaging approach, characterized by its remarkable accessibility, cost-effectiveness, and outstanding patient compliance. The population usually is financially demanding, so TCS is an affordable choice [25,26,27]. In particular, TCS has demonstrated its efficacy in establishing a correlation between the hyper-echogenicity region in the SN and the incidence of PD [28,29]. Recognizing the significance of TCS in PD management, numerous international guidelines have been extensively documented [30,31,32]. TCS imaging can reveal hyper-echogenicity regions within the SN in the midbrain, and regions characterized by an enlarged echogenic area in the SN, measuring between 0.20 and 0.25 cm^2^, occur in approximately 90% of PD. These imaging diagnosis methods can be considered as TCS-based PD diagnosis [27].

In clinical practice, the assessment of the SN region within the midbrain using TCS holds significant importance in the imaging-based diagnosis of PD and serves as a critical component in organizing the whole diagnostic workflow for PD [27,33]. However, the conventional practice of TCS-based diagnosis is entirely reliant on manual procedures, which presents two major drawbacks. First, it is highly time-consuming, which not only imposes considerable clinical burdens, especially in view of the growing demands due to the rapidly expanding aging population, but also necessitates experienced clinicians who are trained in the hyper-echogenicity regions of the SN in the midbrain to achieve accurate assessment results. This challenge could be overwhelming in resource-demanding regions, including developing and underdeveloped countries. Second, the conventional practice lies in the inherent subjectivity of manual assessment processes, which tends to introduce biases into the downstream grading and assessment of PD. Therefore, in the current era of artificial intelligence (AI) in medicine, deep learning-based PD diagnostic algorithms not only alleviate the burden on doctors but also reduce errors caused by subjectivity. Thus, there is an urgent need for an automated, efficient, and objective technique to perform TCS-based PD assessment, which is the first motivation of the research.

In this regard, several methods based on deep learning, especially convolutional neural networks (CNNs), have been applied to explore image-based examinations of PD using different modalities [34,35,36,37]. In 2019, Sivaranjini et al. developed a pre-trained AlexNet model via transfer learning on T2-weighted MR images to classify PD against healthy controls (HCs) using a dataset of 182 subjects, which achieved an accuracy of 88.9% [38]. In the same year, Manzanera et al. proposed an optimized approach employing Conv3D, batch normalization, and non-linearity within a 3D CNN framework. Their method was implemented on a dataset of 310 Fluorodeoxyglucose PET (FDG-PET) scans to classify PD patients against HCs, achieving an accuracy rate of 86.0% [39]. In 2020, Chakraborty et al. proposed a CNN architecture based on 3T T1-weighted MRI scans of 406 subjects acquired from the Parkinson’s Progression Markers Initiative (PPMI) database and achieved an overall accuracy of about 95.3% [37]. Zhao et al. proposed an architecture of a convolutional neural network and a greedy algorithm based on diffusion MRI acquired from a 3T scanner and achieved an accuracy of 80.7% using a PD dataset containing 432 observations [40]. Vyas et al. tested a 2D CNN model and a 3D CNN model and acquired 88.9% accuracy using 318 MRI scans [41]. Notably, one study employed a TCS dataset. In 2020, Shen et al. proposed an improved Deep Polynomial Network (DPN) algorithm, incorporating empirical kernel mapping (EKM), and evaluated it on a TCS dataset of 153 samples. Their method achieved an accuracy of approximately 86.95% [42]. Over the past decade, the aforementioned studies have emphasized the significance of leveraging deep learning techniques in the realm of automated PD diagnosis in patients.

Nevertheless, the contemporary era of AI has placed a growing emphasis on recruiting larger sample sizes for effective learning and including an independent test for model generalizability assessment. The only relevant AI research on TCS-based PD classification in the literature was conducted by Shen et al. However, the authors acknowledged that the sample size was limited because of the practical difficulty of obtaining large datasets in the field of neuroimaging science; therefore, the reported results may still suffer from model overfitting, and hence, their developed models may not be capable of providing sufficient values from clinical implementation perspectives. Moreover, their study did not include an independent testing set for assessing model generalizability, probably because of the insufficient sample size. Further studies using large sample sizes are warranted, which is the second motivation of the present research.

To address these challenges, this study aimed to develop and evaluate a novel deep neural network called dual-channel CNXV2-DANet using a large cohort of 1176 TCS images. The key contributions of the present work are three-fold. First, we employed a large, up-to-date TCS dataset to allow for more effective learning during model development and provide an independent testing set for assessing model generalizability using a naïve dataset. Second, for the first time, we integrated the attention mechanism module from the DANet network into the ConvNeXt V2 architecture, for the sake of enhancing the spatial attention of the network to place greater emphasis on the midbrain and SN regions during model development. Third, we investigated the impacts of single-channel and dual-channel inputs on the network’s learning effectiveness using both left and right sides of TCS images from each of the enrolled subjects, which has not been explored in the current body of literature. The present study is novel in nature, and the findings are expected to offer the community insights into AI-empowered TCS-based workflow for PD assessment, potentially expanding the capacity of TCS imaging for automated population-wide PD screening in community settings towards the smart-aging era.

## 2. Materials and Methods

### 2.1. Data

#### 2.1.1. Data Acquisition

A total of 1176 TCS images from 588 subjects (each subject had a pair of TCS images; there were 307 TCS-based PD patients and 281 TCS-based non-PD patients) were retrospectively enrolled from Beijing Tiantan Hospital. This study was approved by the Ethics Board of Beiing Tiantan Hospital, Capita Medical University (No. KT2022-015-04). Informed consent was obtained from all the participants. Ethical approval was obtained from the Human Subject Ethics Sub-committee (HSESC) of the Hong Kong Polytechnic University (HSEARS20231102004). The original TCS images were obtained using a Canon Aplio i900 i-series ultrasound system (Canon Medical Systems Corporation, Otawara, Tochigi, Japan) equipped with an i6CX1 convex array transducer (center frequency = 2.6 MHz). An experienced physician manipulated the ultrasound probe over the left and right temporal windows of each subject, capturing an optimal ultrasound imaging frame of both sides that depicted the representative butterfly-shaped midbrain structures. All the TCS assessments were performed by two highly experienced physicians with over 10 years of experience, utilizing a TCS-based diagnostic approach. Patients diagnosed with PD using the TCS-based approach from either one side of the TCS images were annotated as TCS-based positive, while those not diagnosed with PD were annotated as TCS-based negative.

#### 2.1.2. Image Pre-Processing

**Figure 1:**
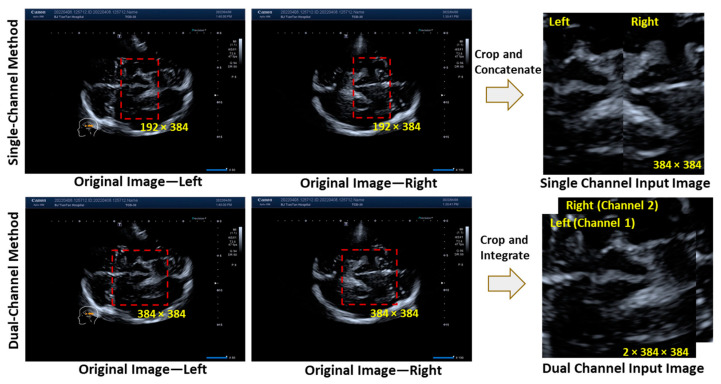
A schematic diagram of the pre-processing approaches for the single-channel input images and dual-channel input images.

Prior to downstream analyses, a series of pre-processing procedures was conducted on the original TCS images of both the left and right sides of the temporal windows. As each subject contained a pair of TCS images (one for the left side and one for the right side), we need to fuse the two images and input them into the model. We developed two pre-processing pipelines, i.e., the single-channel method and the dual-channel method, and analyzed the performance of the studied neural networks. Figure 1 illustrates a schematic diagram for these two methods.

In the single-channel approach, all original TCS images with a resolution of 1280 × 960 pixels were cropped to a size of 192 × 384 pixels, focusing on the midbrain regions as the region of interest (ROI) for both the left (see Figure 1, showing the single-channel method, original image—left) and right (see Figure 1, showing the single-channel method, original image—right) sides of the images. These cropped images were then directly concatenated to create the single-channel input image of 384 × 384 pixels.

For the dual-channel method, the left (see Figure 1, showing the dual-channel method, original image—left) and right (see Figure 1, showing the dual-channel method, original image—right) sides of the original TCS images for each subject were cropped to 384 × 384 pixels as the ROI. These images were then integrated into two separate channels as the dual-channel input image. Both the single-channel and dual-channel methods effectively localized the midbrain region within the ROI while minimizing irrelevant information from other structures, thereby facilitating enhanced learning during subsequent model development.

### 2.2. Deep Learning Neural Networks

In this study, we proposed a neural network called CNXV2-DANet, which leverages the superiority of ConvNext v2 [43] while integrating the attention mechanism module from DANet [44] for the development of the auto-classification model. The attention mechanism model from DANet was added for the sake of promoting the network’s attention toward the midbrain and hyper-echogenicity regions of the SN during model development. The details of the networks are presented below.

#### 2.2.1. ConvNext v2

ConvNext V2 [43] is a convolutional neural network (CNN) architecture that builds on ConvNeXt proposed by Facebook AI Research (FAIR), incorporating the advantages of ResNet [45] and EfficientNet [46]. ConvNext V2 introduces the following innovative components: a fully convolutional masked autoencoder (FCMAE) and a global response normalization (GRN). The FCMAE framework is a CNN-based self-supervised learning method. Its concept involves randomly masking certain regions on input images and then allowing the model to reconstruct the masked portions. This approach compels the model to learn both the global and local features of the images, thereby enhancing its generalization capability. The GRN layer is a novel CNN layer that normalizes feature maps across channels with the purpose of enhancing feature competition among channels by ensuring normalization.

#### 2.2.2. DANet

**Figure 2:**
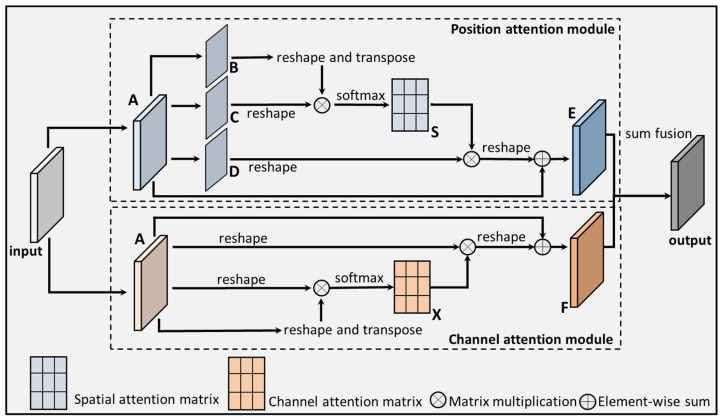
The DANet structure.

DANet is a dual attention network designed to capture global feature dependencies in both spatial and channel dimensions, thereby enhancing the model’s attention weights for crucial locations or channels and improving the model’s performance [44]. Figure 2 visualizes the structure of DANet.

In the position attention module, feature map A undergoes initial processing through 3 convolutional layers to generate 3 feature maps (B, C, and D), which are subsequently reshaped. The transposed form of reshaped feature map B is then multiplied by reshaped feature map C, and the resulting product is subjected to a softmax operation to obtain the spatial attention map (78S). Moreover, reshaped feature map D is multiplied by the transposed S through matrix multiplication, scaled by the factor α, and reshaped back to its original form. Finally, reshaped feature map D is added to A, yielding the ultimate output E. Throughout this process, the weight scaling coefficient α is iteratively learned and adjusted to achieve the optimal value. In the channel attention module, feature map A is reshaped and transposed, and the resulting feature maps are multiplied together. The softmax operation is then applied to calculate channel attention map X. Subsequently, the transposed X is multiplied by reshaped feature map A using matrix multiplication. This product is scaled by the coefficient β and reshaped back to its original form. Finally, the reshaped result is added to A, yielding the final output F. The factor β is iteratively learned to obtain more suitable weights during the training process. To obtain a superior pixel-level prediction of feature representation, the outputs of the two attention modules are combined and aggregated by sum fusion [44].

#### 2.2.3. The Proposed CNXV2-DANet

**Figure 3:**
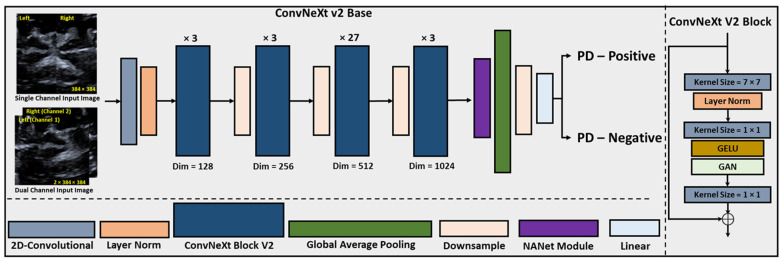
The architecture of CNXV2-DANet.

Figure 3 displays the architecture of CNXV2-DANet, which consists of the basis of ConvNext v2 and the attention mechanism module of DANet. Initially, the network receives the pre-processed images either from the single-channel method or the dual-channel method. Each input undergoes a 2D convolution and Layer Norm operation. Subsequently, the ConvNeXt V2 block is applied in 4 cycles, with a pattern of 3, 3, 27, and 3 convolutional layers, respectively, along with a downsampling step. The DANet module is inserted immediately before the global average pooling layer. Further, a downsampling process is performed, followed by a fully connected layer, generating the classification output.

### 2.3. Model Development and Comparison

#### 2.3.1. Model Development

The entire dataset (*n* = 588) with 1176 TCS images was randomly divided into training, validation, and testing sets via a randomization seed, at an approximate ratio of 70% (*n* = 403), 15% (*n* = 95), 15% (*n* = 90), respectively. In the training set, CNXV2-DANet was utilized to develop a classification model under 5-fold cross-validation for the single-channel method and dual-channel method separately. Referring to Figure 1, a single channel input image contains a direct merge of left-side and right-side cropped TCS images as a single input to the network, whereas a dual channel input image contains two separate cropped TCS images as two separated inputs to the network.

For each of the two methods, 5 randomization seeds were deployed to obtain 5 sets of results for the sake of obtaining an averaged performance value for a fair model evaluation across different patient sub-populations. The approach of multiple randomized stratifications is commonly adopted [47,48]. An averaged performance of each model was reported in this study to assess model stability and to improve the validity of the findings by mitigating sampling bias stemming from random data partitioning. The training process was carried out on an in-house service machine equipped with an Nvidia RTX A6000 GPU card. For the CNXV2-DANet model parameter setting on the training dataset, the model was trained for 50 epochs with a learning rate of 0.00001. The cross-entropy loss function and a weight decay of 0.05 were utilized, while a batch size of 8 was used. Our models operated within the PyCharm (version 2021.3.1) integrated development environment (IDE), and all our experiments utilize CUDA version 12.5.40.

#### 2.3.2. Model Comparison

In order to assess the superiority of the proposed CNXV2-DANet model over the existing state-of-the-art networks, ConvNeXt V2, ConvNeXt [49], and Swin Transformer [50] were individually employed for model comparisons. All the networks were trained using the same training dataset and pre-processing methods, incorporating 5 random seeds and 5-fold cross-validation.

### 2.4. Evaluation Metrics

All the developed models were evaluated on the same testing set; a confusion matrix and several evaluation parameters were applied in this study. For the confusion matrix, four elements were defined as follows: True Positive (TP), False Positive (FP), False Negative (FN), and True Negative (TN). For the evaluation parameters, accuracy, precision, recall, *F*1-score, and area under the ROC curve (AUC) were calculated [51]. The relevant definitions and equations are described below.

Accuracy measures the percentage of correctly classified samples among all samples. In multi-classification tasks, macro-averaged accuracy and micro-averaged accuracy are often used to assess performance. Accuracy is defined by Equation (1) below:

(1) $$Accuracy=\frac{\left(TP+TN\right)}{\left(TP+TN+FP+FN\right)}$$

Precision, also called the positive predictive value, measures the accuracy of positive predictions generated by a model. It calculates the ratio of TP predictions to the total number of predicted positive samples. A higher precision indicates that the model has a lower rate of FPs, making its positive predictions more reliable. This helps minimize incorrect identifications and reduces unnecessary actions or interventions based on FPs. Precision is defined by Equation (2) below:

(2) $$Precision=\frac{TP}{\left(TP+FP\right)}$$

The recall rate quantifies the proportion of TP samples identified by a model out of all the true samples. A higher recall rate indicates that the model is capable of accurately identifying TP samples, thereby demonstrating superior discriminatory ability. It helps us understand the ability of the model to capture all maize disease cases, ensuring that the model does not miss any potential disease instances. Recall is defined by Equation (3) below:

(3) $$Recall=\frac{TP}{\left(TP+FN\right)}$$

The *F*1 score assesses the trade-off between accuracy and recall in a model’s performance. It provides a comprehensive evaluation of how well the model strikes a balance between accurately classifying samples and capturing all relevant instances. A higher *F*1 score signifies that the model has achieved a better equilibrium between accuracy and recall, indicating its proficiency in correctly identifying diseases and ensuring that no instances are overlooked. The *F*1-score is defined by Equation (4) below:

(4) $$F1-Score=\frac{2*(Precision*Recall)}{\left(Precision*Recall\right)}$$

AUC is a crucial metric for evaluating the performance of binary classification models. It measures the area under the ROC curve, which ranges from 0 to 1. A higher AUC indicates better model performance. To calculate AUC, the ROC curve was constructed using the predicted probabilities from the model and the true labels, and the area under the curve was computed. In addition, the DeLong method [52] was also employed to conduct a performance comparative analysis between the proposed CNXV2-DANet model and the other models, yielding calculations for covariance and *p*-values.

## 3. Results

### 3.1. Single-Channel vs. Dual-Channel Methods

#### 3.1.1. CNXV2-DANet

**Figure 4:**
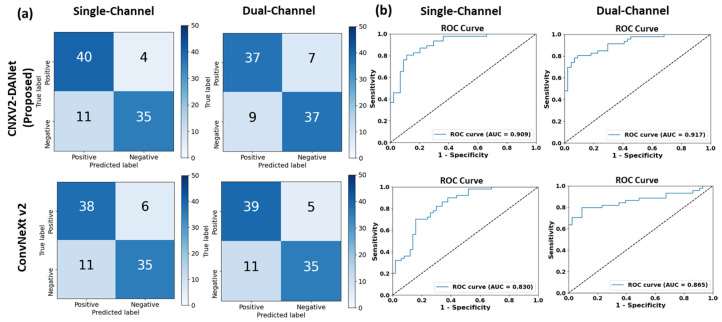
Classification results of CNXV2-DANet and ConvNeXt V2 on the testing set for single-channel and dual-channel methods: (**a**) confusion matrix and (**b**) ROC curve.

Table 1 summarizes the quantitative comparisons of the proposed CNXV2-DANet model between the single-channel and dual-channel pre-processing methods for TCS-based PD classification on the testing dataset. The results of two pre-processing methods were obtained using five randomization seeds under five-fold cross-validation; the averaged evaluation metrics and standard deviation (SD) are reported. Compared with the single-channel method, the dual-channel approach resulted in remarkably higher classification performance, achieving averaged values for accuracy of 0.839 ± 0.028 (single-channel: 0.784 ± 0.037), precision of 0.849 ± 0.014 (single-channel: 0.817 ± 0.090), recall of 0.845 ± 0.043 (single-channel: 0.748 ± 0.093), *F*1-score of 0.820 ± 0.034 (single-channel: 0.773 ± 0.037), and AUC of 0.906 ± 0.013 (single-channel: 0.861 ± 0.047). Figure 4 shows more details of the results from one of the five randomization seeds.

#### 3.1.2. ConvNeXt V2

An ablation experiment was conducted to remove the attention mechanism modules of DANet from the proposed CNXV2-DANet model. Table 2 summarizes the quantitative comparisons of the performance of ConvNeXt V2 between the single-channel and dual-channel pre-processing methods.

All the results were obtained through five-fold cross-validation using five randomization seeds on the same testing dataset, and the averaged values were recorded. Similar to the proposed CNXV2-DANet model, ConvNeXt V2 exhibited superior performance in the dual-channel method compared with the single-channel approach. The dual-channel approach led to remarkably improved classification performance of ConvNeXt V2, achieving averaged values for accuracy of 0.822 ± 0.073 (single-channel: 0.791 ± 0.041), precision of 0.858 ± 0.104 (single-channel: 0.775 ± 0.059), *F*1-score of 0.798 ± 0.074 (single-channel: 0.789 ± 0.046), and AUC of 0.896 ± 0.040 (single-channel: 0.856 ± 0.023), despite its slightly lower averaged recall of 0.775 ± 0.069 (single-channel: 0.813 ± 0.099).

It is worth noting that both CNXV2-DANet and ConvNeXt V2 achieved greater classification performance in the dual-channel setting compared with the single-channel setting (Table 1 and Table 2). Furthermore, the proposed CNXV2-DANet model generally outperformed the ConvNeXt V2 model in the averaged metrics for accuracy of 0.839 ± 0.028 (ConvNeXt V2: 0.822 ± 0.073), recall of 0.845 ± 0.043 (ConvNeXt V2: 0.775 ± 0.069), *F*1-score of 0.820 ± 0.034 (ConvNeXt V2: 0.798 ± 0.074), and AUC of 0.906 ± 0.013 (ConvNeXt V2: 0.896 ± 0.040), despite its slightly lower averaged precision of 0.849 ± 0.014 (ConvNeXt V2: 0.858 ± 0.104).

### 3.2. CNXV2-DANet vs. State-of-the-Art Networks on the Dual-Channel Setting

**Figure 5:**
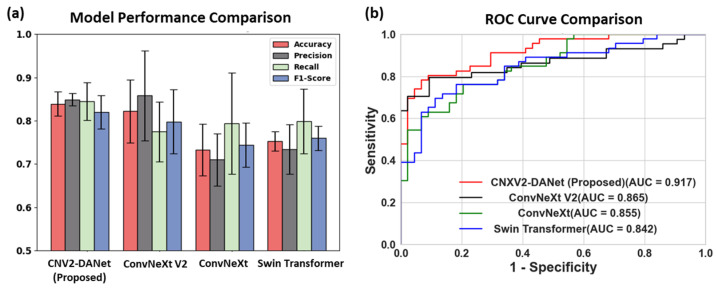
Comparison of the performance of the four studied models on the testing dataset (**a**) in terms of the averaged metrics of accuracy, precision, recall, and *F*1-score with the corresponding standard deviations across the 5 randomizations, and (**b**) in terms of ROC curves under one of the randomization seeds.

Table 3 summarizes the classification performance of the proposed CNXV2-DANet model, in comparison to ConvNeXt V2, ConvNeXt, and Swin Transformer. Among all the comparison networks, the proposed CNXV2-DANet model generally achieved the best classification performance, yielding averaged values for accuracy of 0.839 ± 0.028, precision of 0.849 ± 0.014, recall of 0.845 ± 0.043, *F*1-score of 0.820 ± 0.034, and AUC of 0.906 ± 0.013. This is followed by the ConvNeXt V2 model, while both ConvNeXt and Swin Transformer were the most under-performing networks, incurring tremendously inferior classification performance than the proposed CNXV2-DANet model. Figure 5 displays a comparison of the performance of the studied models on the testing dataset under the dual-channel setting, including the averaged metrics of accuracy, precision, recall, and *F*1-score with the corresponding standard deviations across the five randomizations (Figure 5a) and ROC curves under one of the randomization seeds (Figure 5b). This figure shows that the proposed CNXV2-DANet model achieved the highest AUC value of 0.917, followed by ConvNext v2 with 0.865, ConvNext with 0.855, and Swin Transformer with 0.842. Furthermore, the *p*-values between CNXV2-DANet and ConvNeXt V2, ConvNeXt, and Swin Transformer are all less than 0.001, indicating that CNXV2-DANet’s test performance significantly differs from the other three models.

## 4. Discussion

TCS-based PD diagnosis assumes a pivotal and indispensable role as the initial and fundamental step toward achieving early PD diagnosis with assessment methods. However, the existing assessment process is labor-intensive, time-consuming, and prone to subjectivity. This highlights the urgent need for an automated, efficient, and objective deep learning-based method to meet the growing demand of the community amid the rapidly growing aging population worldwide. Despite the numerous advantages of TCS, such as its non-invasive, radiation-free, rapid, low-cost, and highly accessible nature, high patient compliance, and ease of operation, there is a severe lack of relevant studies focusing on AI-driven TCS-based diagnosis. In light of this, we proposed CNXV2-DANet combined with the dual-channel approach for automated TCS-based PD classification. Our results indicated that the dual-channel approach yielded a great classification performance compared with the single-channel method, and the proposed CNXV2-DANet model outperformed the other comparison networks in a wide range of evaluation aspects. Thus, CNXV2-DANet potentially provides the community with a favorable and automated method for TCS-based PD assessment in the future.

Aligned with the present study, previous studies [28,42,53] conducted over the past decade have consistently emphasized the significance of TCS-based PD diagnosis, which has contributed valuable insights for the research community. Nonetheless, there is an increasing need to leverage the prosperous era of AI and large datasets for streamlining TCS-based PD diagnosis. In recent years, there has been a paucity of studies employing TCS imaging in PD classification-related studies, as shown in Table 4. Furthermore, the dataset we employed was comparatively larger. In 2020, Shen et al. proposed the deep polynomial network (DPN), the deep neural mapping large margin distribution machine (DNMLDM) algorithm, and the dropout and pruning empirical-kernel mapping-deep polynomial network (D-P-EKN-DPN) algorithm [42] for classification of PD with TCS images. Their method achieved an accuracy of 86.95%, sensitivity of 85.77%, specificity of 87.16%, positive predictive value (PPV) of 84.81%, negative predictive value (NPV) of 86.82%, and *F*1-score of 0.86. Their model was trained on a small cohort of fewer than 200 samples, while our study used a large cohort of 588 patients with 1176 TCS images. Their reported results are based on a single random train/test split, which may introduce sampling bias. Additionally, because of the limited sample size, an independent dataset was not available for validation. In contrast, our study adopted a larger cohort of data and conducted five randomization samplings for train/test partitioning to obtain an averaged performance, and the models were evaluated on an independent dataset, thereby offering a greater degree of model inference for naïve datasets in the real world. As shown in Table 1, by leveraging the dual-channel mode and employing the proposed CNXV2-DANet model, we achieved an average accuracy of 83.9% ± 2.8%, precision of 84.9% ± 1.4%, recall of 84.5% ± 4.3%, *F*1-score of 82.0% ± 3.4%, and AUC of 0.906 ± 0.013, compared with other comparing deep learning methods.

In terms of the impacts of single-channel and dual-channel settings, the performance of CNXV2-DANet and ConvNeXt V2 were investigated and analyzed. The findings revealed that the performance of the dual-channel configuration remarkably outperformed that of the single-channel configuration, by CNXV2-DANet, with accuracy (83.9% ± 2.8% vs. 78.4% ± 3.7%), precision (84.9% ± 1.4% vs. 81.7% ± 9.0%), recall (84.5% ± 4.3% vs. 74.8% ± 9.3%), *F*1-score (82.0% ± 3.8% vs. 77.3% ± 3.7%), and AUC (0.906 ± 0.013 vs. 0.861 ± 0.047) referred in Table 1, and by ConvNeXt V2, with accuracy (82.2% 7.3% vs. 79.1% ± 4.1%), precision (85.8% ± 10.4% vs. 77.5% ± 5.9%), *F*1-score (79.8% ± 7.4% vs. 78.9% ± 4.6%), and AUC (0.896 ± 0.040 vs. 0.856 ± 0.023), as shown in Table 2. We speculated that the superior performance of the dual-channel in TCS-based PD diagnosis could be attributed to the enhanced information and richer image information input provided by the dual-channel, which enriches the model’s understanding despite the potential presence of irrelevant or ineffective information. In theory, it is easy to understand why excessive redundancy in input data would have detrimental impacts on model performance. However, excessively compressed data, such as those in the single-channel method, can also adversely affect the model’s classification accuracy. Therefore, it is essential to consider a comprehensive approach that encompasses the selection of appropriate and efficient preprocessing methods.

Furthermore, the proposed CNXV2-DANet model introduces a novel enhancement by incorporating the DANet module with spatial and dual-attention mechanisms. The performance of the CNXV2-DANet model was compared with ConvNeXt V2 through an ablation experiment. The results consistently demonstrated that the proposed CNXV2-DANet model outperformed the ConvNeXt V2 model in both dual-channel and single-channel methods. On the grounds that the hyper-echogenicity in the SN region serves as a prominent marker for potential PD [20], the spatial attention mechanism from DANet was employed to enhance the model’s focus on these critical regions locally within the SN region of the midbrain. We speculated that this may be one of the possible reasons explaining the superiority of the proposed CNXV2-DANet model over the ConvNeXt V2, because the separated channels embedded in DANet may facilitate the complementary learning of the model between the left-side midbrain image and the right-side midbrain image of subjects who may exhibit hyper-echogenicity on their left-side, right-side, or both sides of their SN in midbrain; this capability is absent without the attention module of DANet.

Lastly, the performance of the proposed CNXV2-DANet model was further compared with several state-of-the-art CNN networks, including ConvNeXt V2, ConvNeXt [49], and Swin Transformer [50], under the dual-channel setting. As shown in Table 3, the results demonstrated that CNXV2-DANet achieved remarkably better overall performance, yielding averaged values for accuracy of 83.9%, recall of 84.5%, *F*1-score of 82.0%, and AUC, compared with other comparing networks. However, it is worth noting that ConvNeXt V2 exhibited a higher precision of 85.8%, surpassing CNXV2-DANet’s precision of 84.9%. Considering the research objective of this study, which aims to achieve effective screening of PD, the recall value may hold greater importance in screening PD patients for early diagnosis. Furthermore, we compared the results of CNXV2-DANet and ConvNeXt V2 as part of the results derived from the ablation experiments.

Despite the encouraging results, the present study has several shortcomings that require further investigation in the future. First, the results of this study were generated with a dataset collected from a single institution and the same scanner vendor, which may limit the generalizability of the benchmarked model in real-world clinical settings. A multi-center study with TCS data acquired from different vendors is warranted in the future in order to further validate the findings of this work. Second, although the sample size of 588 (1176 images) in this study was considerably larger than those in previous studies, where the sample sizes ranged from 40 to 130, further investigations using a larger cohort are preferred in the context of deep learning. Third, because of the retrospective nature of this study, although this study recruited highly experienced physicians (>10 years) in ultrasound imaging for generating the reference annotations, there may exist intra- or inter-rater variabilities in the dataset. Therefore, addressing this issue—for example, by expanding the dataset, incorporating multi-center approaches, involving more experienced physicians, and integrating multimodal analyses with a broader range of patient data—in a prospective study is essential for promoting the widespread adoption of the developed model in clinical practice.

## 5. Conclusions

The proposed CNXV2-DANet model, which is configurated based on ConvNeXt V2 and the DANet module, outperformed several existing state-of-the-art networks including ConvNeXt V2, ConvNeXt, and Swin Transformer. Furthermore, the dual-channel approach yielded a remarkably greater PD classification performance than the single-channel method. The findings of this study demonstrated that the proposed CNXV2-DANet model combined with the dual-channel setting has the potential to provide the community with an automated and effective tool for early PD screening using non-invasive, radiation-free, low-cost TCS scanning. Upon resolving the abovementioned shortcomings, the findings of this study may provide valuable insights for clinical practitioners to implement effective and efficient TCS-based PD screening in the smart-aging era, ultimately benefiting countless sufferers worldwide in the long run.

## Figures and Tables

**Table 1:** Performance of CNXV2-DANet in both single-channel and dual-channel settings.

Method		Accuracy	Precision	Recall	*F*1-Score	AUC
Single-channel	Seed1	0.767	0.871	0.614	0.720	0.884
	Seed2	0.789	0.772	0.791	0.782	0.868
	Seed3	0.733	0.679	0.864	0.760	0.784
	Seed4	0.800	0.865	0.711	0.780	0.861
	Seed5	0.833	0.897	0.760	0.823	0.909
	Average	0.784	0.817	0.748	0.773	0.861
	SD	0.037	0.090	0.093	0.037	0.047
Dual-channel	Seed1	0.867	0.833	0.909	0.870	0.924
	Seed2	0.861	0.863	0.862	0.785	0.898
	Seed3	0.844	0.841	0.841	0.841	0.898
	Seed4	0.800	0.865	0.811	0.780	0.894
	Seed5	0.823	0.841	0.804	0.822	0.917
	Average	0.839	0.849	0.845	0.820	0.906
	SD	0.028	0.014	0.043	0.038	0.013

**Table 2:** Performance of ConvNeXt V2 in both single-channel and dual-channel settings.

Method		Accuracy	Precision	Recall	*F*1-Score	AUC
**Single-channel**	Seed1	0.744	0.763	0.674	0.716	0.852
	Seed2	0.844	0.787	0.902	0.841	0.884
	Seed3	0.756	0.690	0.909	0.784	0.838
	Seed4	0.800	0.783	0.818	0.800	0.876
	Seed5	0.811	0.854	0.761	0.805	0.830
	**Average**	0.791	0.775	**0.813**	0.789	0.856
	**SD**	0.041	0.059	0.099	0.046	0.023
**Dual-channel**	Seed1	0.911	0.972	0.837	0.900	0.946
	Seed2	0.811	0.875	0.683	0.767	0.919
	Seed3	0.856	0.878	0.818	0.847	0.903
	Seed4	0.711	0.688	0. 750	0.717	0.847
	Seed5	0.822	0.875	0.761	0.761	0.865
	**Average**	**0.822**	**0.858**	0.775	**0.798**	**0.896**
	**SD**	0.073	0.104	0.069	0.074	0.040

**Table 3:** Quantitative performance of the proposed CNXV2-DANet model and three state-of-the-art networks (ConvNeXt V2, ConvNeXt, and Swin Transformer).

Model	Accuracy (SD)	Precision (SD)	Recall (SD)	*F*1-Score (SD)	AUC (SD)
CNXV2-DANet (proposed)	0.839 (0.028)	0.849 (0.014)	0.845 (0.043)	0.820 (0.038)	0.906 (0.013)
ConvNeXt V2	0.822 (0.073)	0.858 (0.104)	0.775(0.069)	0.798 (0.074)	0.896 (0.040)
ConvNeXt	0.733 (0.045)	0.710 (0.060)	0.794 (0.117)	0.744 (0.051)	0.842 (0.016)
Swin Transformer	0.753 (0.022)	0.734 (0.057)	0.799 (0.075)	0.760 (0.028)	0.834 (0.030)

**Table 4:** PD classification performance comparison with the related-studies in recent years.

Researcher	Year	Dataset	Modality	Method	Accuracy (%)	With an Independent Test Set
Sivaranjini et al. [38]	2019	182	MRI	AlexNet + transfer learning	88.9	No
Manzanera et al. [39]	2019	310	PET	CNN	86.0	**Yes**
Chakraborty et al. [37]	2020	406	MRI	CNN	95.3	No
Shen et al. [42]	2020	153	**TCS**	Deep polynomial network	86.9	No
Zhao et al. [40]	2022	432	MRI	3D CNN	80.7	**Yes**
Our study	2024	**588**	**TCS**	CNXV2-DANet	**83.9**	**Yes**

## Data Availability

The datasets analyzed during the current study are not publicly available due to patients privacy protection purposes, but are available from the corresponding author on reasonable request.
